# Sharing a whole-/total-body [18F]FDG-PET/CT dataset with CT-derived segmentations: an ENHANCE.PET initiative

**DOI:** 10.21203/rs.3.rs-7169062/v2

**Published:** 2025-08-05

**Authors:** Daria Ferrara, Manuel Pires, Sebastian Gutschmayer, Josef Yu, Yasser G. Abdelhafez, Elisabetta Abenavoli, Ramsey D. Badawi, Abhijit J. Chaudhari, Moon S. Chen, Simon R. Cherry, Armin Frille, Barbara K. Geist, Stefan Gruenert, Marcus Hacker, Swen Hesse, Teresa Kerkhoff, Pia Linder, Johanna Pappisch, Smilla Pusitz, Osama A. Raslan, Ivo Rausch, Siba P. Raychaudhuri, Osama Sabri, Fabian Schmidt, Roberto Sciagrà, Benjamin Spencer, Guobao Wang, Hubert Wirtz, Thomas Beyer, Lalith Kumar Shiyam Sundar

**Affiliations:** QIMP Team, Medical University of Vienna, Vienna, Austria; QIMP Team, Medical University of Vienna, Vienna, Austria; QIMP Team, Medical University of Vienna, Vienna, Austria; QIMP Team, Medical University of Vienna, Vienna, Austria. Division of Nuclear Medicine, Medical University of Vienna, Department of Biomedical Imaging and Image-guided Therapy, Vienna, Austria; Department of Radiology, University of California Davis, Sacramento, California, USA; Division of Nuclear Medicine, Azienda Ospedaliero Universitaria Careggi, Florence, Italy; Department of Radiology, University of California Davis, Sacramento, California, USA; Department of Radiology, University of California Davis, Sacramento, California, USA; Comprehensive Cancer Center, University of California Davis, Sacramento, California, USA; Department of Radiology, University of California Davis, Sacramento, California, USA; Department of Respiratory Medicine, University Hospital Leipzig, Leipzig, Germany; Division of Nuclear Medicine, Medical University of Vienna, Department of Biomedical Imaging and Image-guided Therapy, Vienna, Austria; Division of Nuclear Medicine, Medical University of Vienna, Department of Biomedical Imaging and Image-guided Therapy, Vienna, Austria; Division of Nuclear Medicine, Medical University of Vienna, Department of Biomedical Imaging and Image-guided Therapy, Vienna, Austria; Department of Nuclear Medicine, University Hospital Leipzig, Leipzig, Germany; Department of Respiratory Medicine, University Hospital Leipzig, Leipzig, Germany; Division of Nuclear Medicine, University of Tübingen, Tübingen, Germany; Department of Respiratory Medicine, University Hospital Leipzig, Leipzig, Germany; Division of Nuclear Medicine, Medical University of Vienna, Department of Biomedical Imaging and Image-guided Therapy, Vienna, Austria; Department of Radiology, University of California Davis, Sacramento, California, USA; QIMP Team, Medical University of Vienna, Vienna, Austria; Department of Medicine and Dermatology, UC Davis School of Medicine, Sacramento, California, USA; Department of Nuclear Medicine, University Hospital Leipzig, Leipzig, Germany; Division of Nuclear Medicine, University of Tübingen, Tübingen, Germany. Werner Siemens Imaging Center, Department of Preclinical Imaging and Radiopharmacy, University of Tübingen, Germany; Division of Nuclear Medicine, Azienda Ospedaliero Universitaria Careggi, Florence, Italy; Department of Radiology, University of California Davis, Sacramento, California, USA; Department of Radiology, University of California Davis, Sacramento, California, USA; Department of Respiratory Medicine, University Hospital Leipzig, Leipzig, Germany; QIMP Team, Medical University of Vienna, Vienna, Austria; QIMP Team, Medical University of Vienna, Vienna, Austria. DIGIT-X Lab, Department of Radiology, LMU Munich, Germany

**Keywords:** [18F]FDG-PET/CT images, anatomical segmentations, open-sourcing

## Abstract

We present a large whole-body and total-body curated dataset of dual-modality 2-deoxy-2-[18F]fluoro-D-glucose (FDG)-Positron Emission Tomography/Computed Tomography (PET/CT) studies, consisting of 1,597 PET/CT images and the corresponding CT-derived segmentations of 130 target regions. This multi-center dataset includes images from individuals without overt disease and patients with different pathologies (lung cancer, lymphoma, and melanoma). Target regions were first automatically segmented from CT images using an in-house software, and subsequently verified and corrected by physicians-in-training. In total, the segmented regions encompass 130 volumes, including abdominal organs, muscles, bones, cardiac subregions, vessels, adipose tissue, and skeletal muscle around the third lumbar vertebra. PET/CT images and corresponding CT-derived segmentations are provided in anonymized NIfTI format. The dataset can be used for deep learning training, validation, or multi-modality image analysis and thus fills an important gap in available resources to advance the use of PET/CT data in clinical management.

## Background & Summary

In recent years, the field of biomedical engineering and medical physics has witnessed an increase in the complexity of data^1^, driven by rapid advancements in imaging technologies. Traditional data analysis methods have become increasingly inadequate to analyse these large data sets to meet the demand for greater diagnostic precision, and the shift toward personalized treatment strategies^2,3^. To support personalised medicine, more efficient, automated approaches capable of processing and interpreting large-scale datasets are needed. Artificial intelligence (AI) and machine learning (ML) have emerged as powerful tools in this context, offering the ability to identify complex patterns and insights that may not be apparent through conventional methods. However, the effectiveness of AI, particularly of deep learning, is heavily dependent on the availability of large, high-quality and heterogeneous datasets, thus requiring extensive training on vast amounts of data to achieve generalizability and robustness^4^.

Combined positron emission tomography (PET) and computed tomography (CT) integrates both anatomical and functional imaging capabilities, making it indispensable for diagnosing, staging, and monitoring diseases, such as oncological disorders^5–7^. Despite the clinical importance of PET/CT datasets, open sourcing of imaging data is hindered by strict regulations, and analyses are often conducted in-house^8^ on limited data. At present, very few nuclear medicine datasets with annotated lesions are publicly available: only 1,014 PET/CT lung cancer, lymphoma, melanoma, and healthy control cases from the AutoPET challenge^9^, and another 845 head and neck cancer cases through the HECKTOR challenge^10^. In contrast, Ma et al^11^ identified over one million open-source non-nuclear medicine datasets, most of which originating from radiology and not segmented, including more than 350,000 from CT scans alone. This restricts the development and validation of computational methods for functional imaging, such as image and tumor segmentation, volumetric analysis (e.g., for body composition assessment^12^), and radiomics.

Recent advancements in PET/CT technology, particularly the shift from single-organ imaging^13^ to total-body PET/CT systems^14,15^, allow for simultaneous imaging of multiple organs, fueling multi-organ analyses^16^ and the exploration of systemic metabolic abnormalities^17,18^. However, the development of reliable AI methods for automated analysis of these complex datasets requires access to comprehensive open-source resources, including both images and high-quality segmentations of anatomical structures, which are critical for applications such as diagnosis, treatment planning^19^, volumetric analysis, and patient-specific dosimetry^20,21^.

In the field of CT imaging alone, few open-source datasets include corresponding anatomical segmentations. Rister et al.^22^ presented a dataset of 140 abdominal, neck-to-pelvis, and whole-body CT images from patients with liver cancer, segmented into six organ regions. The WORD dataset^23^ comprises 170 abdominal CT images, primarily from prostate, cervical, or rectal cancer cases, along with the segmentations of 16 abdominal organs. These studies, however, are limited in the number of available CT images and segmented regions, and they do not extensively cover different pathologies. More recently, Koitka et al. introduced the Sparsely Annotated Region and Organ Segmentation (SAROS) dataset^24^, which consists of 900 abdominal, thoracic, or whole-body CT images from various pathologies. This work focused on 13 semantic body regions and six body parts, including annotations for every fifth image slice. Similar scope and scale were achieved in the AbdomenCT-1k study^25^, which focused on the liver, kidneys, spleen, and pancreas segmentations, and the comprehensive TotalSegmentator dataset^26^, with CT images of the abdomen, pelvis, or thorax segmented into a total of 104 regions of interest.

However, these datasets are limited in scope, often focusing on specific body regions rather than total-body imaging. It is understood that CT images alone are sufficient for many applications, such as volumetric analysis for body composition^12^ or the delineation of organs at risk in radiotherapy treatment planning^27^. In other applications, however, the functional information from PET imaging is essential as it provides complementary insights into disease mechanisms that CT alone cannot offer. For example, in pathological settings, [18F]FDG-uptake can help track disease progression by detecting systemic changes in metabolism, such as those seen in patients with infections^28,29^, chronic inflammation^30^, metabolic syndrome^31^ or cancer-associated cachexia^32–34^. In studies involving healthy cohorts, longitudinal [18F]FDG PET/CT imaging allows for monitoring metabolic activity in participants and how it changes with aging or other factors^35–37^. Also, a more complete understanding of normal physiological metabolism would help identify deviations that may signal early stages of disease^18^. While the aforementioned AutoPET^9^ and HECKTOR^10^ challenges provide large PET/CT datasets, they focus on segmentations of pathological tissues but ignore healthy anatomical regions.

**Figure 1:**
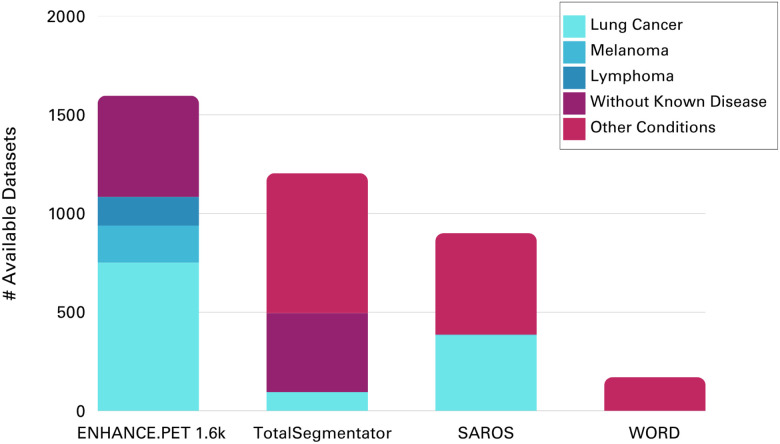
Comparison of clinical indications of cases included in the ENHANCE.PET 1.6k dataset and other open-source CT images and CT-derived segmentations (TotalSegmentator^26^, SAROS^24^ and WORD^23^). The clinical indications for cases within the TotalSegmentator dataset were derived from the non-imaging parameters provided as a CSV file (https://zenodo.org/records/8367088).

In the present study, we address the limited availability of open source PET/CT images with segmented tissues as part of our ENHANCE.PET^38^ initiative, which aims to facilitate the sharing of open-source tools and datasets to support research within the PET community. We curated a large [18F]FDG PET/CT dataset with anatomical segmentations fully verified by human readers. This dataset includes 1,597 whole-body and total-body PET/CT scans, along with corresponding CT-derived segmentations of 130 non-pathological tissues per scan. The initial segmentations were generated using our in-house tool, MOOSE^39^, for automatic CT segmentation and were manually verified and corrected using 3D Slicer^40^, a software platform for image analysis. The data include contributions from the LuCaPET consortium (grant number ERAPerMed_324, *“Clinical decision support for predicting cachexia in cancer patients using hybrid PET/CT imaging”*) and from the AutoPET Challenge^9^, whose images and lesion segmentations were already available as open-source on The Cancer Imaging Archive^41^. Focused mainly on the oncological cases of lung cancer, melanoma, and lymphoma (Fig. 1), the ENHANCE.PET 1.6k dataset also includes participants without known disease. The dataset is provided in anonymized NIfTI format to ensure patient privacy, along with demographic details and CT and PET acquisition parameters as non-imaging metadata.

**Figure 2:**
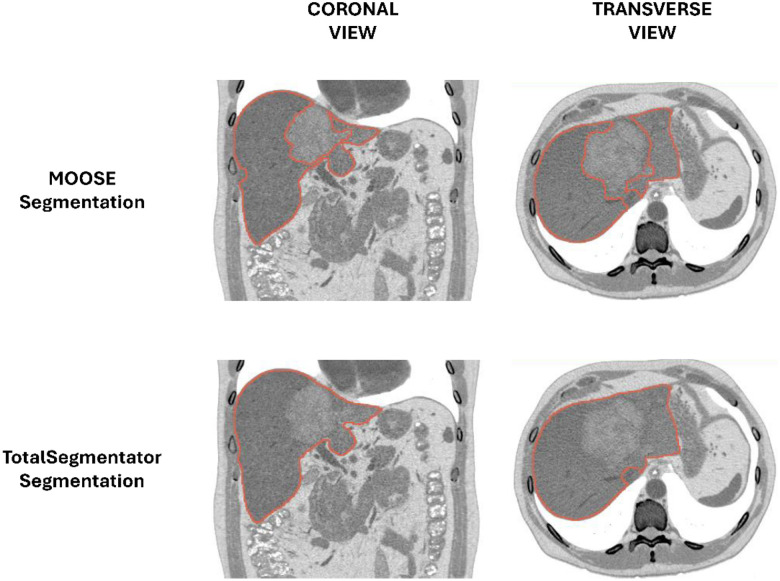
Example of liver segmentation from the “CTLiver” sample data of the 3D Slicer^40^ software. Segmentations were performed using MOOSE^39^, our in-house tool for automatic CT segmentation trained with the ENHANCE.PET 1.6k dataset, as well as TotalSegmentator^26^. Both models accurately segmented the liver volume. However, MOOSE excluded the large liver lesion from the segmentation. In contrast, the output from TotalSegmentator included both healthy and pathological tissue. The ability of MOOSE to differentiate small non-/malignant tissue in low-contrast CT images remains to be studied.

Compared to other publicly available datasets, ENHANCE.PET 1.6k uniquely focuses on the segmentations of organ volumes while avoiding pathological tissues (e.g., tumors, Fig. 2). This dataset is particularly suited for training models aimed at automatic image segmentation and identification of healthy tissues. We believe that the open availability of this dataset will advance the differential understanding of healthy and pathological tissues in computational medicine. This comprehensive resource is now available to facilitate future research and advanced data analysis in whole-body PET/CT imaging. We anticipate that applications such as developing and validating deep learning algorithms for automated data analysis and studies on disease-related systemic abnormalities will greatly benefit from this high-quality data collection.

## Methods

### Data collection

**Figure 3:**
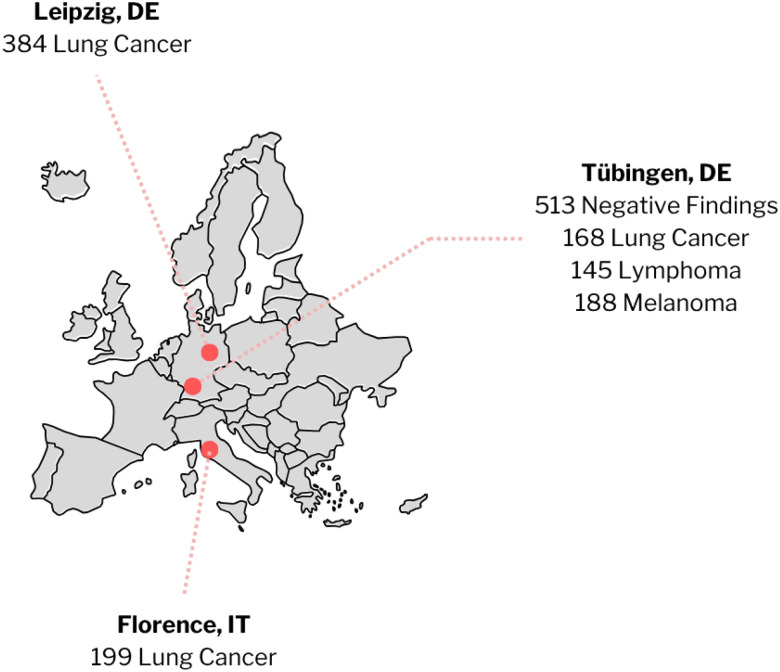
Geographic distributions and clinical indications of the ENHANCE.PET 1.6k dataset. Red dots on the map represent the three clinical facilities of University of Tübingen, Germany; University Hospital Leipzig, Germany; and Azienda Ospedaliero Universitaria Careggi, Italy.

The ENHANCE.PET 1.6k dataset was acquired in accordance with the guidelines set forth in the Declaration of Helsinki. Images were acquired between 1999 and 2022 from various institutions and studies, summarized in Fig. 3: the open-source dataset AutoPET^9^, the University Hospital Leipzig in Germany (IRB: 259/18-ek) and the Azienda Ospedaliero Universitaria Careggi in Italy (IRB: 21306_oss) as part of the LuCaPET consortium.

Participant demographics across the different clinical conditions are summarized in Table 1.

### Imaging Protocols

Three different PET/CT systems were used for image acquisition at the participating medical centres: Siemens Biograph mCT (N = 1398), Philips Gemini TF (N = 179), and GE Healthcare Discovery MI (N = 20). At all three sites, diagnostic CT scans were acquired with X-ray tube voltages between 100 kVp and 140 kVp, and CT data were reconstructed with a slice thickness between 1 mm and 5 mm. Details on the CT reconstruction parameters are provided in Table 2.

Participants were asked to fast for 6 hours before the examinations and were scanned in the supine position, with arms up. Each subject underwent a static PET acquisition following an intravenous injection of [18F]FDG (314 ± 48 MBq). Uptake times varied across the three sites, with an average of (68 ± 21) minutes post-injection. PET images were reconstructed with attenuation and scatter corrections applied using the corresponding CT data.

Details on the CT and PET acquisition parameters are reported for each participant as non-imaging parameters in the available spreadsheet files. The download link is provided in the Data Records section.

### Segmentations and Data Processing

**Figure 4:**
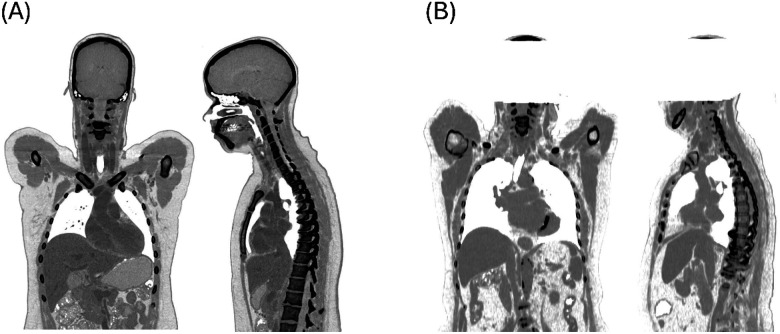
Coronal and sagittal views of (A) an original CT image from the AutoPET Challenge^9^ and (B) an anonymized CT image, defaced, from the Azienda Ospedaliero Universitaria Careggi, Italy. Defacing of the PET/CT images was performed as an additional measure for complete anonymization of patients prior to data open-sourcing.

PET/CT images were retrieved in anonymized DICOM format from the participants and centralized at the Medical University of Vienna. The metadata were used to extract relevant information about the CT and PET acquisition protocols as well as essential demographic details of the participants. For subsequent analysis and segmentation, all data were converted to NIfTI format using the *dcm2niix* DICOM to NIfTI converter^42^. To ensure that participants could not be visually identified from their CT images^43–45^, both the PET and CT images from the Azienda Ospedaliero Universitaria Careggi and the University Hospital Leipzig were edited: in the PET images, voxels between the upper part of the skull segmentation and the bottom of the brain, within a cylinder of 16 voxels in the z-direction, were set to zero (Fig. 4B). Similarly, the corresponding CT region was set to −1000 Hounsfield Units to simulate air. The images from the AutoPET challenge were left unedited as per their version available online.

To maximize efficiency and accelerate the workflow, the processing of the entire ENHANCE.PET 1.6k dataset was done serially: automatic segmentation of the CT images, manual refinement of the derived labels, and retraining of the original segmentation models, according to the following scheme. Our in-house developed software, MOOSE^39^, was first used for the automatic segmentation of 384 lung cancer images from the University Hospital Leipzig. The resulting segmentations were manually refined by 10 medical students using the 3D Slicer image analysis software^40^. For each dataset, a student was randomly assigned to verify and correct the segmentations, addressing possible systematic errors such as inaccuracies at anatomical borders of target regions, mislabelling between left and right regions, or misclassification of regions with similar intensities on CT. A second student was then tasked with reviewing the first student’s work and correcting any remaining mistakes. Once both students agreed on the final version of the dataset, it was reviewed by a radiology resident and a nuclear medicine resident. The PET images overlapped with the corresponding CT were used to exclude tumor volumes from the segmentation of various abdominal organs.

**Figure 5:**
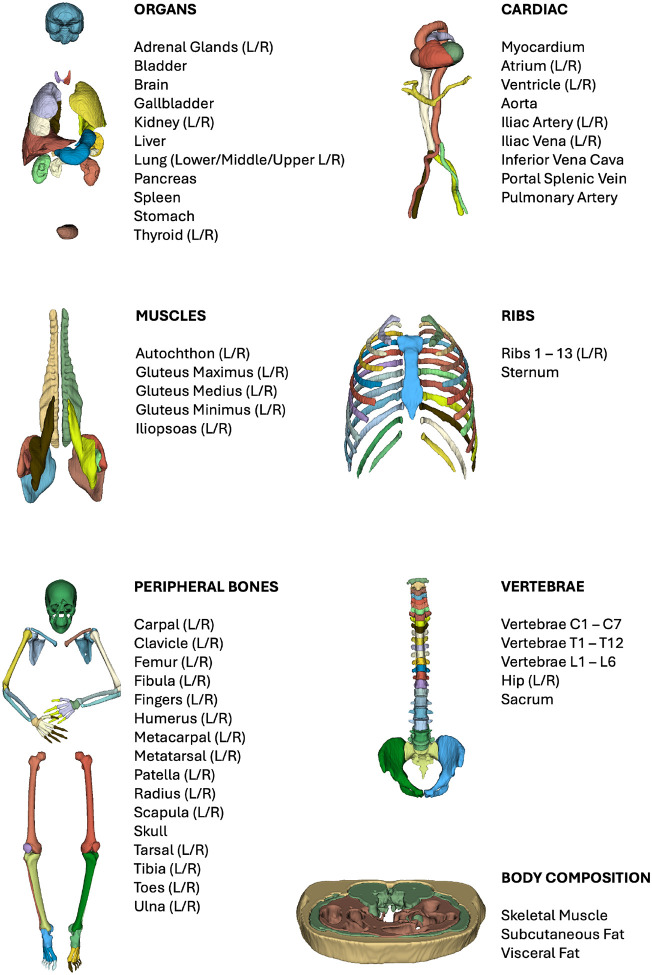
Complete list of segmented target regions per dataset. L = left; R = right.

These preliminary segmentations were divided into seven different classes, as shown in Fig. 5: organs, cardiac, muscles, ribs, peripheral bones, vertebrae, and body composition around the L3 vertebra area. These masks were then used to retrain our models using nnU-Net^46^. Details on the retraining process are provided in the Technical Validation section.

The newly trained models were subsequently used for the segmentation of the second dataset, originating from Azienda Ospedaliero Universitaria Careggi, and again underwent manual refinement and quality control described above. The same workflow of automatic segmentation, manual refinement, and model retraining was then applied to the remaining images from the AutoPET^9^ open-source dataset. In the case of the AutoPET data, since the corresponding lesion segmentations were available online, they were taken as ground truth and directly subtracted from the organ segmentations without the need for manual correction.

To maximize data variability and ensure robust performance across different scanning conditions, we included in our training dataset 86 additional total-body CT images (30F/56M, 53 ± 15 years, 87 ± 19 kg, 172 ± 9 cm) from the University of California Davis, California (USA), acquired on a United Imaging uEXPLORER PET/CT system. The segmentations were generated using the same workflow as for the other datasets. Their inclusion helped account for different acquisition protocols (arms-down positioning) and provided broader representation of physiological variations, as they covered various inflammatory and pathological conditions, such as head and neck cancer (N = 5), arthritis (N = 43), genito-urinary cancer (N = 9), and healthy controls (N = 29). While these cases cannot currently be shared due to privacy restrictions, we are working to make them publicly available in the future.

**Figure 6:**
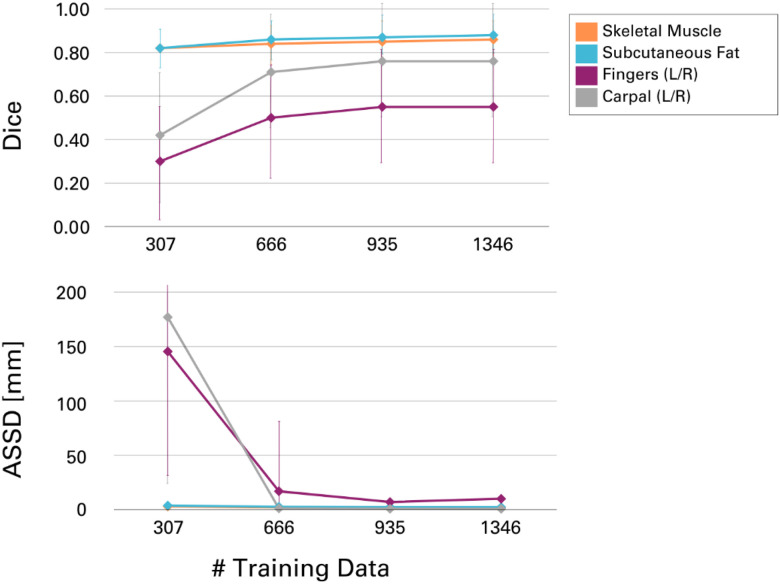
Performance of the CT-segmentation models on selected target regions as a function of the training dataset size. Performance was evaluated on 20% of the ENHANCE.PET 1.6k dataset (N=337), as described in the Technical Validation section. As the training dataset size increased, DICE scores for segmentation improved, primarily due to the correction of systematic segmentation errors, such as left/right misclassification in the hands and the inclusion of missing regions in the “skeletal muscle” segmentation. Similarly, the average symmetric surface distance (ASSD) decreased as the dataset size grew.

At each stage of retraining, the size of the training data increased, improving model performance. This iterative process allowed for efficient verification and faster corrections by the medical students without compromising precision, especially in regions where systematic errors had been identified and were hindering the manual correction process. Segmentation of the fingers and hand bones showed the most significant improvement as the training dataset grew (Fig. 6). In the first round of training, several instances of left-right misclassification were identified, especially when the hands were crossed over the abdomen or above the head. However, this issue progressively improved with each retraining step. Another improvement achieved through more extensive training data was the automatic inclusion of the quadratus lumborum muscle in the “skeletal muscle” label for body composition, which had previously been missing and required manual correction (Fig. 6).

### Data Records

The PET/CT images and corresponding segmentations from Azienda Ospedaliero Universitaria Careggi, University Hospital Leipzig, and the AutoPET^9^ dataset are stored on Amazon Web Services (AWS, https://aws.amazon.com/) and can be downloaded either directly following MOOSE^39^ installation via command line, as described in the Code Availability section, or at the following link:

https://enhance-pet.s3.eu-central-1.amazonaws.com/enhance-pet-1_6k/enhance-pet-1_6k.zip

**Figure 7:**
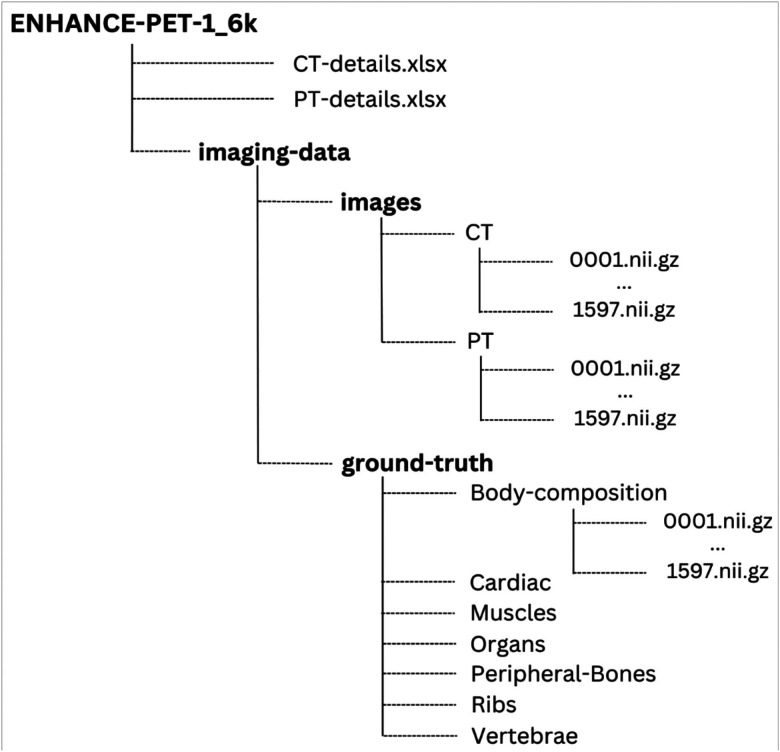
Folder structure of the ENHANCE.PET 1.6k dataset. Each image and segmentation are provided in anonymized NIfTI format and named with ascending unique IDs from 0001.nii.gz to 1597.nii.gz. For each participant, the CT image, PET image, and segmentations of cardiac subregions and vessels, muscles, organs, peripheral bones, ribs, vertebrae, and body composition (including skeletal muscle and adipose tissue) around the L3 vertebra region are provided.

The imaging data are stored in three separate folders containing the CT images, PET images, and ground truth segmentations, respectively. Within the segmentations folder, there are seven subfolders corresponding to different segmentation classes: “Body-Composition,” “Cardiac,” “Muscles,” “Organs,” “Peripheral Bones,” “Ribs,” and “Vertebrae.” Each folder contains NIfTI data files, named sequentially from 0001.nii.gz to 1597.nii.gz. The directory structure of the ENHANCE.PET 1.6k dataset is shown in Fig. 7.

A JSON file containing the complete list of segmentations and their corresponding intensities within the multi-class files is available for download at the following link:

https://enhance-pet.s3.eu-central-1.amazonaws.com/enhance-pet-1_6k/labels.json

In addition to the imaging data, non-imaging information is provided in two spreadsheet files. The *CT-details.xlsx* file contains details on the CT acquisition parameters (e.g., PET/CT system manufacturer and model, kVp, filter type, convolutional kernel, axial pixel size, slice thickness, and focal spot size) for each participant. The *PT-details.xlsx* file provides the corresponding demographic information (e.g., clinical indication, sex, age, weight, height) as well as PET acquisition parameters (e.g., injected activity, acquisition date and time, radioactivity injection details, image units, slope, intercept, system model and manufacturer).

### Technical Validation

We used the ENHANCE.PET 1.6k dataset with the additional 86-image contribution from the University of California, Davis to develop a deep learning-based method for the automatic segmentation of CT scans: 80% of the images and corresponding labels were sampled from the total with a stratified sampling, thus ensuring that the original proportion of data by medical facility and clinical condition remained unchanged. The 1346 selected imaging data served as a training dataset for multiple segmentation models targeting different anatomical regions, including bones of the limbs and skull, thoracic cage bones, vertebrae and sacrum, major abdominal organs, lower back muscles, cardiac tissues, and body composition around the L3 vertebra. A detailed list of regions segmented by each model is shown in Fig. 5.

Prior to training, all images and labels were resampled with SimpleITK (https://simpleitk.org/about.html) from the original resolution to a voxel spacing of 1.5 × 1.5 × 1.5 mm using B-spline interpolation. This provided a resolution high enough to segment fine structures on the CT images, while considering the computational burden of training. The models were trained using nnU-Net^46^, a state-of-the-art self-configuring framework based on the U-Net architecture for semantic segmentation. The training process was performed over 2000 epochs.

**Figure 8:**
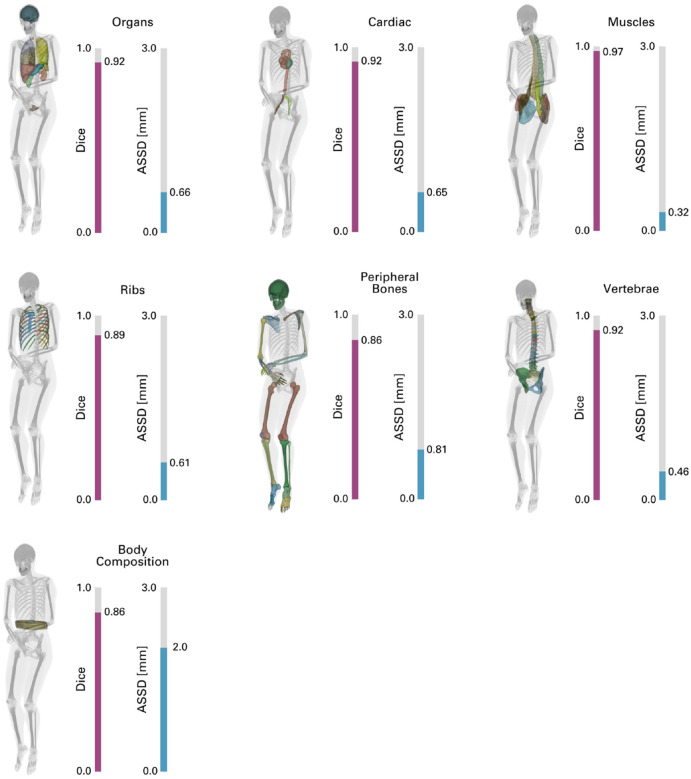
Mean Dice scores and Average Symmetric Surface Distance (ASSD) per available segmentation model between reference labels of the test dataset (N=337, 20% of the total ENHANCE.PET 1.6k) and the labels resulting from MOOSE^39^ prediction.

To assess the model performance, we tested all segmentation models on the remaining 20% of the ENHANCE.PET 1.6k. Segmentation accuracy was evaluated using the Dice Similarity Coefficient (DSC) to quantify the overlap between predicted segmentations and the reference labels and with the Average Symmetric Surface Distance (ASSD)^47^ to estimate the average distance between surface voxels of the reference labels and the automated segmentation. The averaged results for the generated models are shown in Fig. 8, and the metrics for each label are reported in Table 4.

All models achieved high accuracy, with mean DSC values exceeding 0.85 across most regions and mean ASSD values below 3 mm in all regions. The “Muscles” model achieved the highest overlap and the lowest prediction error, with an average DSC of 0.97 ± 0.02 and an ASSD of 0.3 ± 0.2 mm (Fig. 8). Cardiac, organ, and vertebrae models also achieved high average DSC values, exceeding 0.90. The peripheral bones model had the lowest performance, with an average DSC of 0.86 ± 0.27 and the highest variation in ASSD, at 0.8 ± 7.2 mm. The lower performance in these regions is likely due to their small size and thin anatomical structures: the digits of the hand had the most significant negative impact on model performance, with some cases of left/right misclassification identified (especially when patients underwent imaging with their hands crossed over the abdomen), resulting in an average DSC of 0.55 ± 0.39 and an ASSD of 10 ± 37 mm (Table 4). Similarly, the segmentation of the metacarpals yielded a DSC of 0.71 ± 0.33 and an ASSD of 5 ± 23 mm. Other regions with lower overlap included the portal and splenic veins (DSC: 0.82 ± 0.18) and the adrenal glands (DSC: 0.82 ± 0.12), most likely due to their low contrast resolution in CT imaging of the test dataset, which makes delineation more challenging. The ribs and body composition models also showed higher variation in ASSD, at 0.6 ± 2.1 mm and 2.0 ± 1.7 mm, respectively.

The ENHANCE.PET 1.6k dataset proved to be satisfactory for training models for automated CT image segmentation. This dataset has the potential to contribute significantly to further advancements in deep learning-based algorithms, including attempts to improve segmentation models performance or the addition of new volumes of interest not covered in the present study. The dual availability of both CT and PET images, together with the inclusion of segmentations for multiple anatomical regions, makes the ENHANCE.PET 1.6k dataset particularly valuable for research focused on diseases that affect multiple organs or systems, such as metabolic disorders or systemic inflammatory diseases^48,49^, or for studies on normal glucose metabolism in healthy tissues. As a limitation, since the segmentations were derived from the CT images, the alignment with the corresponding PET images may be compromised in cases of significant patient motion, which was not systematically assessed in this study.

We hope that this open-source dataset will accelerate developments in medical imaging, ultimately contributing to the advancement of personalized medicine and more effective clinical decision-making.

### Usage Notes

The contributions of the Azienda Ospedaliero-Universitaria Careggi and the University Hospital Leipzig to the ENHANCE.PET 1.6k dataset are licensed under the Creative Commons Attribution 4.0 International License (CC BY 4.0). Data from the AutoPET^9^ Challenge are licensed under the Creative Commons Attribution-NonCommercial 4.0 International License (CC BY-NC 4.0). All imaging data are presented in NIfTI format, ensuring participants’ privacy while allowing for easy use in further analysis. This format can be opened with most visualization software, including 3D Slicer (https://www.slicer.org/) and ITK-SNAP (http://www.itksnap.org/pmwiki/pmwiki.php). DICOM to NIfTI conversion was performed using *dcm2nii*^42^, and all image processing was conducted using Python.

## Figures and Tables

**Table 1:** Demographics and clinical details of the participants included in the study.

Partner University	Clinical Condition	#	Sex	Age [years]	Weight [kg]	Height [cm]
Azienda Ospedaliero Universitaria Careggi, IT	Lung Cancer	199	72F / 127M	71 ± 10	72 ± 15	168 ± 9
University Hospital Leipzig, DE	Lung Cancer	384	110F / 274M	65 ± 11	76 ± 16	172 ± 9
Open-source dataset AutoPET^9^	Lung Cancer	168	65F / 103M	60 ± 15	80 ± 18	173± 10
	Lymphoma	145	69F / 76M	57 ± 18	79 ± 19	173± 11
	Melanoma	188	77F / 111M	60 ± 16	79 ± 20	172 ± 9
	Negative Findings	513	233F / 280M	60 ± 16	79 ± 18	171 ± 11

**Table 2:** Summary of CT systems and reconstruction parameters of the ENHANCE.PET 1.6k dataset.

Partner University	Azienda Ospedaliero Universitaria Careggi, IT N = 199	University Hospital Leipzig, DE N = 384	Open-source dataset AutoPET^9^ N = 1014
PET/CT System Manufacturer	GE Medical System (19)	Siemens	Siemens
	Philips (180)		
System Model	Discovery MI (19)	Biograph 16 (207)	Biograph 128 (900)
	Gemini TF TOF 16 (180)	Sensation 16 (177)	SOMATOM
			Definition (114)
kVp	120 (196)	120	100 (2)
	140 (3)		120 (938)
			140 (74)
Filter Type	N/A	NONE	FLAT
Convolutional Kernel	N/A	B10f (325)	I30f (31)
	STANDARD (19)	B31f (56)	I31f (471)
		B40f (3)	B30f (24)
			B31f (488)
Axial Pixel Size (mm)	0.98–1.37	0.98	0.69–0.98
Slice Thickness (mm)	3.75 (19)	2 (36)	1 (52)
	5 (180)	3 (347)	2 (141)
			3 (821)
Focal Spot Size (mm)	N/A	0.7 (330)	1.2
		1.2 (54)	

**Table 4:** Mean Dices and Average Symmetric Surface Distance (ASSD) per segmented volume between the reference labels of the test dataset (N = 337, 20% of the total ENHANCE.PET 1.6k) and the labels resulting from the AI prediction. Left and right regions were merged for this analysis.

Model	Regions	Mean Dice ± St Dev	Mean ASSD ± St Dev [mm]
Organs	Adrenal Glands	0.82 ± 0.12	0.5 ± 0.5
	Bladder	0.90 ± 0.18	1.0 ± 2.0
	Brain	0.96 ± 0.13	0.6 ± 0.4
	Gallbladder	0.88 ± 0.15	0.8 ± 1.7
	Kidney	0.96 ± 0.05	0.5 ± 0.7
	Liver	0.98 ± 0.03	0.6 ± 0.5
	Lung Lower Lobe	0.95 ± 0.12	0.6 ± 1.4
	Lung Middle Lobe	0.94 ± 0.09	0.8 ± 1.8
	Lung Upper Lobe	0.97 ± 0.05	0.9 ± 0.6
	Pancreas	0.90 ± 0.08	0.7 ± 0.7
	Spleen	0.96 ± 0.08	0.7 ± 0.8
	Stomach	0.95 ± 0.06	0.7 ± 1.0
	Thyroid	0.87 ± 0.19	0.6 ± 0.8
Cardiac	Myocardium	0.92 ± 0.05	0.5 ± 0.4
	Atrium	0.96 ± 0.03	0.5 ± 0.4
	Ventricle	0.96 ± 0.03	0.5 ± 0.4
	Aorta	0.95 ± 0.02	0.5 ± 0.2
	Iliac Artery	0.90 ± 0.09	0.6 ± 1.9
	Iliac Vena	0.92 ± 0.07	0.8 ± 1.8
	Inferior Vena Cava	0.92 ± 0.06	0.6 ± 0.5
	Portal Splenic Vein	0.82 ± 0.18	0.9 ± 1.8
	Pulmonary Artery	0.94 ± 0.05	0.8 ± 0.6
Muscles	Autochthon	0.98 ± 0.01	0.3 ± 0.2
	Gluteus Maximus	0.98 ± 0.01	0.3 ± 0.2
	Gluteus Medius	0.98 ± 0.01	0.3 ± 0.2
	Gluteus Minimus	0.96 ± 0.03	0.3 ± 0.2
	Iliopsoas	0.97 ± 0.02	0.3 ± 0.2
Ribs	Rib 1	0.86 ± 0.12	0.5 ± 0.6
	Rib 2	0.88 ± 0.12	0.4 ± 0.8
	Rib 3	0.88 ± 0.11	0.4 ± 0.6
	Rib 4	0.90 ± 0.10	0.4 ± 0.9
	Rib 5	0.90 ± 0.09	0.5 ± 2.0
	Rib 6	0.91 ± 0.09	0.5 ± 1.8
	Rib 7	0.91 ± 0.09	0.6 ± 2.4
	Rib 8	0.91 ± 0.09	0.6 ± 2.3
	Rib 9	0.90 ± 0.10	0.7 ± 1.9
	Rib 10	0.90 ± 0.11	0.8 ± 2.7
	Rib 11	0.89 ± 0.14	0.9 ± 3.2
	Rib 12	0.86 ± 0.18	0.9 ± 3.7
	Sternum	0.94 ± 0.03	0.5 ± 1.1
Vertebrae	Vertebra C1	0.89 ± 0.10	0.5 ± 0.7
	Vertebra C2	0.92 ± 0.09	0.4 ± 0.4
	Vertebra C3	0.91 ± 0.09	0.4 ± 0.4
	Vertebra C4	0.90 ± 0.09	0.4 ± 0.5
	Vertebra C5	0.90 ± 0.11	0.4 ± 0.4
	Vertebra C6	0.90 ± 0.09	0.4 ± 0.4
	Vertebra C7	0.91 ± 0.09	0.4 ± 0.6
	Vertebra T1	0.93 ± 0.09	0.4 ± 0.7
	Vertebra T2	0.93 ± 0.10	0.4 ± 0.9
	Vertebra T3	0.92 ± 0.10	0.4 ± 0.9
	Vertebra T4	0.93 ± 0.09	0.4 ± 0.9
	Vertebra T5	0.93 ± 0.07	0.4 ± 0.5
	Vertebra T6	0.93 ± 0.08	0.4 ± 0.6
	Vertebra T7	0.93 ± 0.09	0.4 ± 1.0
	Vertebra T8	0.94 ± 0.08	0.4 ± 0.9
	Vertebra T9	0.94 ± 0.08	0.5 ± 1.0
	Vertebra T10	0.94 ± 0.10	0.5 ± 1.2
	Vertebra T11	0.94 ± 0.11	0.5 ± 1.4
	Vertebra T12	0.94 ± 0.12	0.6 ± 1.7
	Vertebra L1	0.93 ± 0.13	0.7 ± 2.2
	Vertebra L2	0.93 ± 0.14	0.7 ± 2.4
	Vertebra L3	0.93 ± 0.15	0.7 ± 2.6
	Vertebra L4	0.93 ± 0.16	0.8 ± 2.6
	Vertebra L5	0.90 ± 0.20	0.6 ± 2.0
	Hip	0.97 ± 0.08	0.3 ± 0.2
	Sacrum	0.96 ± 0.08	0.3 ± 0.2
Peripheral Bones	Carpal	0.76 ± 0.33	0.8 ± 0.6
	Clavicle	0.91 ± 0.16	0.5 ± 2.1
	Femur	0.96 ± 0.15	0.3 ± 0.6
	Fibula	0.93 ± 0.19	0.3 ± 0.5
	Fingers	0.55 ± 0.39	10.0 ± 37.5
	Humerus	0.94 ± 0.15	0.4 ± 1.0
	Metacarpal	0.71 ± 0.33	5.2 ± 22.7
	Metatarsal	0.92 ± 0.17	0.8 ± 4.1
	Patella	0.92 ± 0.19	0.5 ± 1.0
	Radius	0.66 ± 0.42	0.5 ± 0.8
	Scapula	0.91 ± 0.16	0.3 ± 0.4
	Skull	0.90 ± 0.16	0.5 ± 0.6
	Tarsal	0.95 ± 0.17	1.4 ± 4.5
	Tibia	0.95 ± 0.16	0.3 ± 0.3
	Toes	0.90 ± 0.15	0.6 ± 1.3
	Ulna	0.68 ± 0.40	0.5 ± 0.6
Body Composition	Skeletal Muscle	0.85 ± 0.08	2.0 ± 1.5
	Subcutaneous Fat	0.87 ± 0.08	2.4 ± 1.8
	Visceral Fat	0.87 ± 0.09	1.5 ± 1.6

## Data Availability

The segmentation software MOOSE and the presented open-source dataset are part of the ENHANCE.PET (https://enhance.pet/) initiative for facilitating data sharing and collaboration within the PET community. In case of downloading and usage of the dataset, please cite the ENHANCE.PET initiative and website. MOOSE code is open-source and available online with extensive documentation, and can be accessed on GitHub at https://github.com/ENHANCE-PET/MOOSE. Following MOOSE installation within a Python environment, the dataset can be downloaded via the command line: < moosez -dtd -dd path/to/download/ >
